# Cytogenetics of Neotropical fishes: Patterns, advances and prospects after five decades of research

**DOI:** 10.1590/1678-4685-GMB-2025-0190

**Published:** 2026-05-22

**Authors:** Mauro Nirchio, Claudio Oliveira

**Affiliations:** 1 Universidad Técnica de Machala, Departamento de Acuicultura, Machala, Ecuador.; 2 Universidade Estadual Paulista (UNESP), Instituto de Biociências, Departamento de Biologia Celular e Molecular, Botucatu, SP, Brazil.

**Keywords:** Neotropical fishes, fish cytogenetics, karyotype evolution, sex chromosomes, repetitive DNA

## Abstract

This review synthesizes the most significant advances and innovations in the cytogenetic study of Neotropical fishes, with emphasis on recurrent chromosomal patterns, evolutionary mechanisms, and the integration of modern genomic tools. The Neotropical region, which harbors more than 12,000 fish species, exhibits not only extraordinary taxonomic richness but also remarkable chromosomal diversity. Chromosomal rearrangements have been associated with speciation and ecological adaptation in both freshwater and marine lineages. Modern techniques, including fluorescence *in situ* hybridization, chromosome painting, and comparative genomic hybridization (CGH), have substantially improved our ability to resolve morphologically indistinguishable species and clarify their evolutionary relationships. When integrated with phylogenetic and genomic approaches, cytogenetics becomes a robust framework for exploring biodiversity and chromosomal evolution within the complex Neotropical fauna. Despite these advances, substantial challenges remain, particularly the scarcity of cytogenetic data for many taxa and regions. Future research, driven by high-throughput genomic technologies, is expected to deepen our understanding of chromosomal evolution and to support conservation efforts for this rich but increasingly threatened fish fauna.

## Introduction

The origins of cytogenetics date back to the 19th century, when the Swiss botanist Carl Nägeli first described filiform structures in the nuclei of plant cells, which he called “transitional cytoblasts”, and are now recognized as chromosomes. The term “chromosome” was coined in 1888 by Heinrich Wilhelm Gottfried von Waldeyer-Hartz, from the Greek *chroma* (color) and *soma* (body), following the development of staining techniques that allowed them to be visualized more clearly (Kannan and Zilfalil, 2009). At the beginning of the 20th century, the chromosomal theory of inheritance proposed independently by Sutton (1903) and Boveri (1902), consolidated the idea that chromosomes carried Mendelian factors, and transformed cytologists into cytogeneticists (Ferguson-Smith, 2015).

Modern cytogenetics underwent a technical revolution in the 1950s. One of the most important advances was the use of colchicine to arrest cells in metaphase by inhibiting mitotic spindle formation, thereby facilitating the observation of condensed chromosomes. This procedure was applied by Joe Hin Tjio and Albert Levan in their landmark 1956 study, in which they accurately determined that the diploid number (2n) of human cells was 46 (Kannan and Zilfalil, 2009).

Another essential advance was the introduction of hypotonic pretreatment, to improve chromosome dispersion. This method showed that exposure of cultured mammalian cells to hypotonic solutions induced cell swelling and allowed better separation of chromosomes (Kannan and Zilfalil, 2009; Ferguson-Smith, 2015; Balajee and Hande, 2018). Although initially tested with aqueous solutions and citrates, the use of potassium chloride (KCl) at a concentration of 0.075 M was consolidated during the 1960s as the standard hypotonic solution, thanks to its ability to achieve an optimal balance between cell expansion and chromosome preservation (Balajee and Hande, 2018).

In this broader historical and conceptual context, fish cytogenetics has become a particularly dynamic field. Since the 1960s, standardized protocols for obtaining mitotic chromosomes from tissues such as kidney, gill and spleen have allowed rapid advances, particularly in South America, a region recognized as a global hotspot of freshwater ichthyofaunal diversity (Oliveira et al., 2009; Artoni et al., 2015). Although many of these cytogenetic techniques were initially developed in mammals and other vertebrates, they were soon adapted to the physiological characteristics of fishes, including adjustments for tissue osmolality, enzymatic digestion, and mitotic indices (Clem et al., 1961; Booke, 1968; Denton, 1973; Wolf and Ahne, 1982).

In the last five decades, the application of classical cytogenetic tools, such as C-banding (Sumner, 1972), Ag-NOR staining (Howell and Black, 1980), and fluorescent *in situ* hybridization (FISH) (Pinkel et al., 1986) together with more recent genomic approaches, has revealed remarkable diversity in 2n, karyotypic formulas, sex chromosome systems and repetitive DNA organization in Neotropical fishes (Artoni et al., 2015; Bello Cioffi et al., 2018; Paim et al., 2018; Wang et al., 2024). This diversity is not merely descriptive, but reflects underlying evolutionary and ecological processes that shape genome architecture over time. Patterns of chromosomal variation reflect the influence of lineage-specific dynamics, and broader factors such as habitat fragmentation, hydrographic isolation and historical dispersal. In the complex and heterogeneous landscapes of South America, riverine barriers and ecological gradients have repeatedly influenced population structure, promoting chromosomal differentiation through genetic drift, local adaptation and restricted gene flow.

Consequently, cytogenetics serves not only as a catalog of structural genomic features but also as a powerful lens through which to explore the mechanisms that generate and maintain biodiversity in Neotropical ichthyofauna. Advances in molecular cytogenetics have demonstrated how chromosomal architecture, especially with respect to repetitive DNA, sex chromosomes, and rDNA sites, can influence lineage diversification and genomic evolution (Toledo and Foresti, 2001; Bello Cioffi et al., 2012). Within this integrative framework, the combination of cytogenetics, genomics, and phylogenetics has become increasingly essential for resolving taxonomic uncertainties, detecting cryptic species, and reconstructing evolutionary trajectories (Dutra et al., 2020; Liehr, 2021). As cytogenetics has moved from a predominantly descriptive science toward a genomic and evolutionary discipline, its role in understanding the generation of biodiversity is more important than ever (Garcia-Sagredo, 2008).

In commemoration of the 70th anniversary of the Brazilian Society of Genetics (SBG), this review synthesizes the principal discoveries and methodological advances in Neotropical fish cytogenetics. This trajectory began with the seminal publication by Bertollo et al. (1978) on the karyotype of *Hoplias lacerdae* in the inaugural volume of the *Brazilian Journal of Genetics* and continues to influence the field today. This review also acknowledges the contributions of researchers in fish cytogenetics with particular recognition to the foundational contributions of renowned Brazilian cytogeneticists, including Dr. Fausto Foresti (Instituto de Biociências, UNESP), Dr. Luis Antônio Carlos Bertollo, Dr. Pedro Manuel Galetti Júnior, Dr. Orlando Moreira Filho (UFSCar), and Dr. Lurdes Foresti de Almeida Toledo (USP), whose pioneering work helped shape fish chromosome research in South America. Within the framework of the SBG, their efforts established a robust academic and scientific legacy spanning the past five decades.

Here we highlight the historical development of fish cytogenetics, its role as an integrative tool in evolutionary biology, and its current relevance in the genomic era. From the earliest descriptions of karyotypes to modern cytogenomics, the study of Neotropical fish chromosomes continues to illuminate the genetic basis of biodiversity and evolutionary change. This genomic and chromosomal diversity must also be understood in the broader evolutionary and biogeographic context of the Neotropical freshwater fish fauna, which is the most species-rich in the world, shaped by complex fluvial dynamics, ecological gradients, and geological history (Albert et al., 2020). 

## Fish cytogenetic techniques

Methodological innovation has been a driving force of cytogenetics. Early studies focused on determining chromosome number and karyotype formulas (e.g., Bertollo et al., 1978) and soon afterward, techniques for identifying specific chromosomal regions were incorporated. Among these, silver nitrate impregnation remains a classic method for detecting active nucleolus organizer regions (Ag-NOR) (Howell and Black, 1980).

**Figure 1 -:**
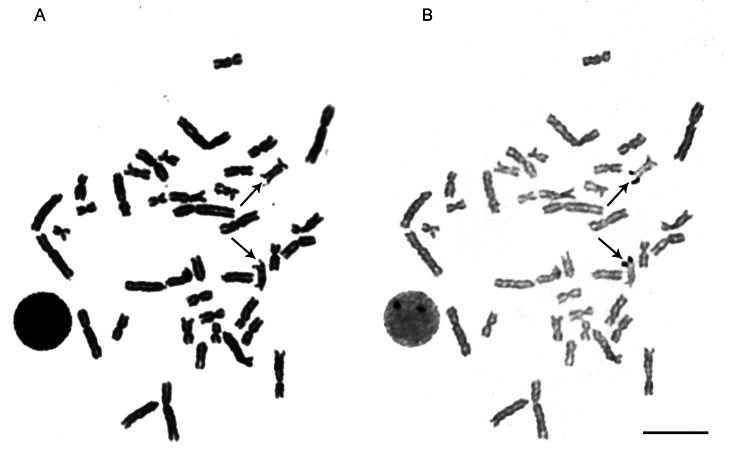
Sequential chromosomal staining in *Gymnothorax* sp. (A) Metaphase plate stained with Giemsa. (B) Same metaphase after Ag-NOR staining, showing active nucleolus organizer regions (NORs) as black signals on a single chromosome pair (arrows). A large interphase nucleus is visible in both panels, displaying two nucleoli corresponding to the NORs. Scale bar = 10 µm. Unpublished data. Image provided by the authors.

An illustrative example is shown in metaphase chromosomes initially stained with Giemsa, revealing a karyotype composed predominantly of biarmed elements. Subsequent silver nitrate staining on the same preparation identified a single pair of NOR-bearing chromosomes with intense terminal signals (Figure 1). A large interphase nucleus adjacent to both metaphases displays two distinct nucleoli, corresponding precisely to the number and position of the NORs observed in metaphase. This demonstrates the transcriptional activity of these regions and exemplifies how Ag-NOR staining provides not only structural but also functional cytogenetic information. Such approaches remain fundamental for characterizing species lacking genomic resources and for establishing cytogenetic baselines in teleosts.

**Figure2 -:**
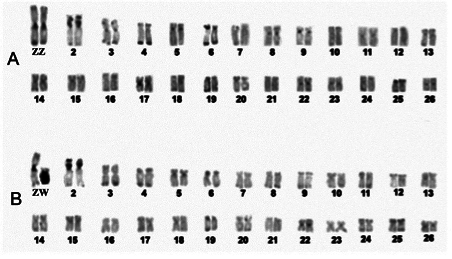
Karyotype of *Triportheus signatus* after application of the C-banding technique. In (A) male, (B) female. The darker regions near the centromeres, telomeres, and the long arm of the W chromosome represent heterochromatic regions rich in satellite sequences. Unpublished data. Image provided by the authors.

Soon after its development, the identification of heterochromatic regions by the C-banding technique (Sumner, 1972) was applied by Park and Grimm (1981) to study the ZW sex chromosome system of the American eel. The method rapidly became a standard in fish cytogenetics, allowing the differentiation of specific chromosome pairs within distinct karyotypes. For example, the sex chromosomes of *Triportheus signatus* are easily distinguishable after C-banding (Figure 2).

**Figure 3 -:**
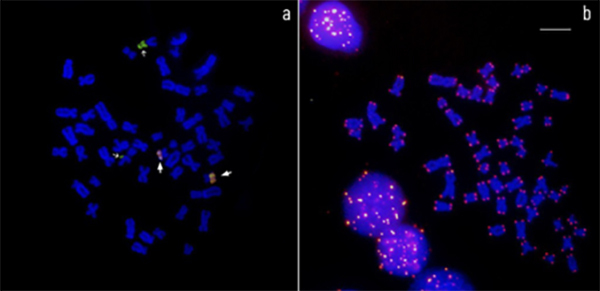
Mitotic metaphase of *Ancistrus clementinae* analyzed by FISH using specific probes for 18S (green) and 5S (red) ribosomal genes (a) and (TTAGGG)n probes (b). Sites of 18S rDNA (marked with thin white arrows) and 5S rDNA (thick white arrows) are observed located on different chromosome pairs, indicating non colocalized distribution of these two types of ribosomal genes. Scale bar: 5 µm. Image provided by the authors.

Methodological advances soon expanded the scope of cytogenetics, enabling not only the accurate determination of chromosome numbers and structural abnormalities, but also the development of more sophisticated molecular tools. One of the most transformative was FISH, introduced in the 1980s (Knoll and Lichter, 2005; Pinkel et al., 1986). This technique uses fluorescently labeled DNA probes that hybridize to complementary sequences, allowing the identification of specific genes or chromosomal regions. For example, ribosomal genes (18S and 5S rRNA), satellite DNA and telomeric repeats can thus be visualized directly on chromosomes (Figure 3).

Whole chromosome painting (WCP) enables the identification of homologous chromosomes and the reconstruction of chromosomal rearrangements using chromosome-specific fluorescent probes. An example of its application to sex chromosome evolution is presented in Section 5.3.

Following the development of FISH, several advanced variants, such as multicolor fluorescence in situ hybridization (mFISH), CGH, and genomic *in situ* hybridization (GISH), further enhanced the resolution of chromosomal analyses. These approaches have proven especially useful for detecting parental contributions in hybrids and for genome-wide comparisons of repetitive DNA among individuals, sexes, or species. Combined with high-throughput sequencing and other genomic tools, these methods have transformed cytogenetics from a descriptive discipline into an integrative science that bridges chromosomal structure and genome-wide information, giving rise to the emerging field of *Chromosomics* (Liehr, 2021).

## Biodiversity

The Neotropical region is home to the richest freshwater fish fauna on Earth, with 6,345 valid freshwater species, according to the most recent and comprehensive ecological assessment (Albert et al., 2025). This number reflects ongoing taxonomic discovery and the remarkable ecological and morphological diversity across South and Central America and the Greater Antilles. While estimates for marine fish diversity in the Neotropical region remain less precise, global data indicate approximately 14,800 marine fish species, although no complete regional inventory is currently available. This taxonomic gap underscores the importance of coordinated efforts to document ichthyofaunal diversity in both continental and coastal ecosystems. The region’s fish diversity encompasses not only extraordinary taxonomic richness, but also a wide range of morphologies, life histories, ecological roles, and biogeographic patterns (Winemiller, 1989; Toussaint et al., 2016). The unique geological history of the region, including the uplift of the Andes and the evolution of large river basins such as the Amazon, Orinoco, and Paraná, has contributed to complex patterns of speciation and endemism, resulting in highly localized faunas (Albert *et al*., 2018; Dagosta and Pinna, 2019).

However, this rich biodiversity is increasingly threatened. Freshwater ecosystems in the Neotropics are under increasing pressure from anthropogenic activities such as dam construction, deforestation, mining, agriculture, urbanization, overfishing, and biological invasions (Pelicice et al., 2021). These factors act synergistically, causing habitat fragmentation, altered hydrological regimes, reduced connectivity, and pollution, all of which contribute to the erosion of fish diversity and ecosystem functioning (Agostinho et al., 2016; Anderson et al., 2018).

The situation is particularly critical in previously pristine regions, such as parts of the Amazon and the Andes, where environmental degradation has accelerated due to weak environmental policies and development pressures. The special issue on Neotropical freshwater fish diversity (*Neotropical Ichthyology*, vol. 19, no. 3) documents a wide range of ecological consequences, from demographic shifts and species extirpations to biotic homogenization and loss of ecosystem services, that underscore the urgency of strengthening conservation and management efforts (Pelicice et al., 2021).

In addition to taxonomic loss, studies also report functional and phylogenetic erosion, which may compromise the ecological resilience and multifunctionality of ecosystems (Leal et al. 2021; Souza et al., 2021). Despite growing awareness, conservation initiatives remain insufficient, and many protected areas do not encompass regions of great diversity (Oliveira et al., 2021). To safeguard the evolutionary legacy and ecological services provided by Neotropical fishes, both freshwater and marine, a shift towards integrated, evidence-based conservation planning is urgently needed.

## Cytogenetic diversity

Neotropical fishes, both freshwater and marine, exhibit some of the greatest karyotypic diversity known among vertebrates, reflecting their enormous taxonomic richness, ecological diversity, and complex evolutionary history. Cytogenetic studies have documented a wide range of 2n, karyotypic formulas, structural polymorphisms and supernumerary chromosomes. This variation ranges from conserved karyotypes to highly rearranged configurations.

Cytogenetic diversification is more pronounced in freshwater fishes than in their marine counterparts. A comparative study of 103 Neotropical species revealed that the modal 2n was 54 in freshwater species, whereas marine species were predominantly characterized by 2n = 48, usually composed of acrocentric chromosomes (Nirchio et al., 2014). In addition, the *fundamental number* (FN) and proportion of biarmed chromosomes were significantly higher in freshwater species, suggesting a higher frequency of fusions, inversions and duplications, possibly favored by habitat fragmentation and geographic barriers limiting gene flow.

Most of the marine fish analyzed (60%) exhibit conservative karyotypes with 2n = 48 and exclusively acrocentric chromosomes. This homogeneity has been attributed to high genetic connectivity, large population sizes, and active migration, which hinder the fixation of chromosomal rearrangements (Soares et al., 2021). However, techniques such as FISH and chromosome banding have revealed dynamic microstructures in marine groups such as Haemulidae, Serranidae and Lutjanidae, all with 2n = 48, but with significant variations in the distribution of ribosomal genes and other repetitive sequences (Galetti et al., 2000; Nirchio et al., 2007; Nirchio *et al*., 2008).

These comparisons not only enrich our understanding of environment-dependent karyotypic evolution but also challenge the classical hypothesis that the ancestral karyotype of teleosts consisted of 48 acrocentrics. Recent evidence suggests that the ancestral pattern may have been more complex, with higher chromosome numbers and a high proportion of biarmed elements (Brum and Galetti, 1997; Nakatani et al., 2007). The analysis presented in this section is primarily based on the comprehensive chromosomal dataset compiled by Arai (2011), which remains a foundational reference for evaluating karyotypic patterns across Neotropical teleost orders. Where relevant, this comparative framework has been enriched with updated cytogenetic and cytogenomic studies to reflect recent methodological advances and taxonomic insights.

An essential consideration for reinterpreting the status of the acrocentric 2n = 48 karyotype is the distinction between homology and chromosomal analogy. This distinction has been emphasized in the theoretical frameworks of karyotype evolution developed by Sankoff and Nadeau (2003), who demonstrated that similar chromosome configurations can arise repeatedly in independent lineages due to structural constraints, mechanical stability, and highly constrained evolutionary trajectories. Under this framework, the recurrence of the acrocentric 2n = 48 karyotype across multiple marine lineages does not by itself constitute evidence of shared ancestry. Instead, this pattern is more consistent with recurrent or convergent chromosomal configurations shaped by population connectivity and selective constraints typical of marine environments. Thus, the prevalence of this karyotype in contemporary marine teleosts should be interpreted cautiously and may reflect recurrent chromosomal outcomes rather than direct retention of an ancestral state. This conceptual framework directly challenges the traditional “karyotype ground plan” proposal and reinforces the hypothesis that the acrocentric 2n = 48 is more consistent with a derived condition in several lineages rather than a universally plesiomorphic state within Actinopterygii.

Within this interpretive framework, Neotropical Mugiliformes provide a particularly illustrative case in which apparent karyotypic conservatism conceals substantial chromosomal and taxonomic diversity**.** Cytogenetic studies of this group have progressed from early reports emphasizing conserved karyotypes to a more nuanced understanding of chromosomal evolution and species delimitation, especially when cytogenetic data are integrated with morphological and molecular evidence.

Early cytogenetic analyses of Venezuelan mugilids revealed striking contrasts between closely related taxa. *Mugil liza* exhibits a karyotype composed of 48 acrocentric chromosomes, consistent with the modal condition traditionally proposed for Mugilidae, whereas *Mugil curema* displays a highly derived karyotype with 2n = 24, composed entirely of bi-armed chromosomes (22 metacentric and two submetacentric pairs) (Nirchio and Cequea, 1998). The diploid number 2n = 48 has been reported in several mugilid species, including *M. cephalus*, *Chelon labrosus*, *Liza aurata*, *L. ramada*, and *M. parsia*, and has long been interpreted as ancestral due to its frequency and the predominance of uniarmed chromosomes (Cataudella and Capanna, 1973; Arai, 2011). This view reinforced the perception of karyotypic conservatism within the family.

However, the identification of markedly divergent karyotypes challenges this interpretation and points to a dynamic chromosomal evolutionary history within Mugilidae. The occurrence of 2n = 24 in Venezuelan populations of *M. curema* and 2n = 28 in populations from Louisiana (LeGrande and Fitzsimons, 1976) suggests that extensive chromosomal restructuring has occurred, most plausibly through Robertsonian fusions. This hypothesis is supported by the larger size of the bi-armed chromosomes in *M. curema* relative to the acrocentric chromosomes of *M. liza*, indicating centric fusion events rather than fissions, a mechanism widely considered more likely in fish chromosomal evolution (Nirchio and Cequea, 1998).

Subsequent comparative analyses further reinforced this scenario. Brazilian populations of *M. curema* exhibit a derived karyotype with 2n = 28, composed predominantly of metacentric and submetacentric chromosomes, whereas *Mugil margaritae* from Margarita Island shows a karyotype with 2n = 24 and similar chromosomal morphology (Menezes et al., 2015). In both cases, the fundamental number (FN = 48) is maintained, supporting the hypothesis that chromosome arm number is more evolutionarily stable than diploid number and underscoring the central role of Robertsonian rearrangements in mugilid karyotype diversification (Nirchio et al., 2005; Rossi et al., 2005).

Other species retain the plesiomorphic condition. *Mugil incilis* exhibits 2n = 48 acrocentric chromosomes and a single NOR-bearing pair, corroborating its basal position within the group (Hett et al., 2011). In contrast, *Mugil rubrioculus*, originally grouped with *M. curema*, displays a distinct karyotype and allozyme profile; together with morphological differences, this evidence justified its recognition as a separate species (Harrison et al., 2007; Nirchio et al., 2007).

The integration of cytogenetics with molecular phylogenetics has proven decisive for resolving cryptic diversity within Mugilidae. A notable example is the identification of *Mugil* sp. O in the eastern Pacific, characterized by a unique karyotype (2n = 46; two metacentric and 44 subtelocentric/acrocentric chromosomes), distinct from all Atlantic forms. Subsequent integrative analyses led to the reassignment of this lineage to *Mugil setosus*, a historically described but poorly documented species whose revalidation was supported by both mitochondrial and cytogenetic evidence (Nirchio et al., 2017; Britzke et al., 2019).

Overall, Mugiliformes exemplify how similar chromosomal configurations, including the widespread 2n = 48 acrocentric condition, may reflect recurrent and convergent rearrangements rather than strict lineage inheritance, reinforcing the need to interpret karyotypic patterns within an explicit evolutionary framework.

### Karyotypic diversity in Neotropical orders

In contrast to the predominantly marine and coastal Mugiliformes discussed above, Neotropical freshwater fishes represent one of the most diverse and evolutionarily complex ichthyofaunas worldwide, shaped by long-term continental isolation, drainage fragmentation, and heterogeneous ecological conditions. This assemblage encompasses a broad array of freshwater lineages with contrasting ecological strategies and genomic architectures. Although 14-16 teleost orders inhabit Neotropical freshwater ecosystems (Albert and Reis, 2011; Reis *et al*., 2016; Betancur-R et al., 2017), cytogenetic coverage remains uneven.

This section focuses on six orders (Osteoglossiformes, Characiformes, Siluriformes, Gymnotiformes, Cyprinodontiformes, and Cichliformes), which collectively capture the phylogenetic, ecological, and genomic breadth of Neotropical freshwater fishes and represent the most extensively characterized groups from a chromosomal perspective. The analysis presented here is primarily based on the comprehensive chromosomal dataset compiled by Arai (2011), which remains a foundational reference for evaluating karyotypic patterns across Neotropical teleost orders. Where relevant, this comparative framework is enriched by recent cytogenetic and cytogenomic studies reflecting methodological and conceptual advances.

*Osteoglossiformes*

As one of the most basal teleost groups, Osteoglossiformes display a wide range of diploid numbers (2n=40-56) and chromosomal formulas across different genera. In the Neotropics, *Arapaima gigas* shows 2n = 56 and FN = 84, with distinct rDNA sites and heterochromatic blocks enriched in microsatellites (Oliveira et al., 2019; Oliveira *et al*., 2020).

Other osteoglossids display contrasting configurations. *Heterotis niloticus* (2n = 40; FN = 76) exhibits a more derived complement dominated by submetacentric and acrocentric elements. *Osteoglossum bicirrhosum* and *O. ferreirai* share 2n = 56 but differ significantly in the proportion of chromosome morphologies. *Scleropages* species (2n = 44-50; FN = 74-84) highlight intrageneric chromosomal evolution.

Additional families contribute further diversity: *Pantodon buchholzi* (Pantodontidae) exhibits 2n = 48 with a balanced mixture of biarmed and acrocentric chromosomes. Mormyrids such as *Gnathonemus petersii* and *Marcusenius brachistius* (2n = 48; FN = 53-55) show predominantly acrocentric karyotypes. Notopterids including *Chitala chitala*, *Xenomystus nigri*, and *Notopterus notopterus* exhibit a conserved pattern (2n = 42; FN = 42-44).

*Characiformes*

Characiformes, one of the most species-rich Neotropical orders, exhibits extensive karyotypic diversity. Although the modal diploid number is 2n = 50-54, reported values range from 36 to 102 (Arai, 2011), reflecting fissions, fusions, and possible polyploid events. Most species show predominant metacentric (M) and submetacentric (SM) chromosomes, yet the proportions of subtelocentric (ST) and acrocentric (A) elements vary widely. The FN ranges from 66 to more than 100 and is often decoupled from 2n due to frequent pericentric inversions and Robertsonian rearrangements.

Genera such as *Astyanax*, *Characidium*, *Leporinus*, and *Triportheus* exhibit marked chromosomal variation at both interspecific and intraspecific levels. The *Astyanax scabripinnis* complex is a classical model of microevolutionary cytogenetics, showing variation in 2n, karyotypic formula, heterochromatin distribution, and frequent B chromosomes (Souza and Moreira-Filho, 1995; Castro et al., 2014). Morphometric divergence across cytotypes further underscores the evolutionary complexity of this group (Mizoguchi and Martins-Santos, 1998).

Within Serrasalmidae, a family including *Serrasalmus*, *Pygocentrus*, *Metynnis*, and *Piaractus*, karyotypic patterns are phylogenetically structured. The ancestral 2n is 54, retained by *Piaractus* and *Colossoma*. In contrast, all reliably analyzed species of *Serrasalmus* and *Pygocentrus* display 2n = 60 with FN = 100-116 (Arai, 2011; Nakayama et al., 2002; Favarato et al., 2021). *Metynnis* shows an increased 2n = 62 attributed to ascending dysploidy via chromosomal fissions. These transitions illustrate coordinated evolution of diploid numbers and karyotype architecture and align with ecological diversification across the family (Favarato *et al*., 2021; Jacobina et al., 2023).

Thus, Characiformes represent a powerful model for investigating chromosomal evolution, genome diversification, and speciation dynamics in freshwater environments.

*Siluriformes*

Siluriformes exhibits remarkable chromosomal heterogeneity among families, although cytogenetic coverage remains uneven.

In Auchenipteridae, most species have 2n = 58 while genera such as *Ageneiosus*, *Tympanopleura*, and *Tetranematichthys* show independent reductions (2n = 52-56), interpreted as centric fusions not shared among lineages. The use of molecular tools, including FISH for mapping rDNA and interstitial telomeric sites (ITS), has revealed cryptic reorganizations valuable for differentiating morphologically similar species (Felicetti, 2023; Casarotto et al., 2024; Kowalski et al., 2024).

Doradidae, previously considered conservative, exhibits considerable diversity in karyotype formulas, NOR phenotypes, and heterochromatin patterns. The ancestral 2n = 58 is maintained in most species although several lines of *Anadoras* (Astrodoradinae) exhibit 2n = 56 derived from independent fusions. C-banding and rDNA mapping have allowed the identification of specific chromosomal signatures for each subfamily, supporting multiple episodes of structural reorganization within the group (Takagui et al., 2021, 2022, 2024).

Trichomycteridae remains one of the least studied clades (<10% of species analyzed). While most cis-Andean species retain 2n = 54, trans-Andean forms show a wider range (2n = 50-64), attributable to geographic fragmentation and independent evolutionary dynamics (Borin and Martins-Santos, 2004; Sato et al., 2004). The historical dependence on conventional techniques underscores the need to expand the use of cytogenomic methodologies in the group.

A recent synthesis shows that cytogenetic variation in Siluriformes ranges from 2n = 40 to more than 130 in Callichthyidae, 2n = 32-62 in Trichomycteridae, 2n = 50 in Scoloplacidae, and 2n = 52-54 in Astroblepidae, reflecting an evolutionary history marked by episodes of ascending and descending dysploidy (Sassi et al., 2024). Within Loricariidae, which includes Hypostominae, Ancistrinae, Loricariinae, and Hypoptopomatinae, variations in 2n, FN, heterochromatin distribution, and rDNA patterns evidence intense activity of fusions, fissions, inversions, and expansions of repetitive elements. The absence of a universal ancestral state, coupled with the rapid ecological radiation of the group, suggests that chromosomal reorganization processes have acted repeatedly and convergently. According to this review, even within well-defined subfamilies, chromosomal trajectories are strongly lineage-specific, reinforcing the need to expand taxonomic sampling and systematically apply advanced molecular techniques to understand the group’s diversification patterns.

*Gymnotiformes*

Gymnotiformes, an exclusively Neotropical order of electric fishes, exhibit remarkable cytogenetic diversity, with 2n ranging from 34 to 74 (Arai, 2011; Suárez et al., 2017). This wide range reflects dynamic karyotypic evolution, characterized by lineage-specific fusions and fissions. In *Gymnotus*, particularly in *G. carapo* and *G. inaequilabiatus*, molecular cytogenetic analyses have uncovered multiple cryptic cytotypes and markedly divergent chromosomal architectures, revealed through techniques such as comparative genomic hybridization (CGH) and WCP (Milhomem et al., 2008; Nagamachi et al., 2010; Machado et al., 2018). These differences, often associated with allopatric distributions, suggest reproductive isolation and incipient speciation. *Rhabdolichops* cf. *eastwardi* possesses the highest 2n recorded for the order (2n = 74), interpreted as a result of lineage-specific chromosomal fission events (Suárez *et al*., 2017).

*Cyprinodontiformes*

Neotropical guppies and killifishes of the order Cyprinodontiformes, particularly the annual representatives of the family Rivulidae, have evolved in highly fragmented and extreme environments, such as seasonal ponds, flooded savannas and high-altitude lakes. These ecological conditions, characterized by low dispersal potential and strong reproductive isolation, favor accelerated rates of karyotypic evolution. Cytogenetic studies have revealed remarkable variation in 2n (30-48), FN, NOR patterns and heterochromatin distribution (do Nascimento et al., 2014).

In this context, *Hypsolebias antenori* stands out for having a karyotype composed of 48 biarmed chromosomes and FN = 96. This condition, attributed to several pericentric inversions, may represent an advanced evolutionary stage. This contrasts with *Cynolebias* species, in which chromosome number has decreased due to centric fusions (García et al., 1993, Scheel, 1972). Another illustrative case is *Rivulus mahdiaensis*, a species native to the Guiana Shield, with 2n=38 and a pair of notably large acrocentric chromosomes, suggesting gradual and continuous restructuring of its karyotype (Suijker and Collier, 2006).

The genus *Austrolebias* represents a unique model for the study of genomic evolution under extreme seasonal conditions. With karyotypes ranging from 2n = 28 to 48, and exceptionally high nuclear DNA content (~6 pg per diploid cell), these species show a strong expansion of transposable elements, high chromosomal instability and zones of natural hybridization that contribute to phenotypic and genomic diversity (García et al., 2022).

In contrast, non-annual killifishes such as *Cyprinodon dearborni* and *Orestias ascotanensis* exhibit structurally stable karyotypes, with genomic repeats (rDNA, histones, small nuclear RNA) located on specific chromosomes. These profiles suggest greater structural conservation and genomic adaptations to extreme environments such as hypersaline or high-altitude waters (Nirchio et al., 2003; Araya-Jaime et al., 2017).

*Cichliformes*

Cichlids constitute a classical model for chromosomal evolution in Neotropical fishes due to their ecological diversity and adaptive radiation. Most species maintain 2n = 48 (the plesiomorphic condition for the group) but show substantial structural variation in chromosomal formulas (Poletto et al., 2010; Hodaňová et al., 2014). Major evolutionary trends include: a) conservation of the ancestral 2n; b) increases in chromosome number via centric fissions; and c) reductions via Robertsonian fusions.

Large metacentrics arising from such fusions often accumulate repetitive DNA, a pattern recurrently associated with cichlid karyotype differentiation (Poletto et al., 2010; Bello Cioffi et al., 2012). Chromosomal inversions can further diversify karyotypes and may contribute to reduced recombination and reproductive isolation by suppressing recombination across rearranged regions (Blumer et al., 2025). 

It is important to note, as discussed earlier in Section 4, that the plesiomorphic condition of 2n = 48 within Cichlidae does not imply that this same diploid number represents the ancestral state of teleosts as a whole. As previously highlighted, similarities in chromosome number can reflect distinct evolutionary trajectories rather than shared ancestry. According to the general models of chromosomal evolution proposed by Sankoff and Nadeau (2000), the recurrent conservation of certain diploid numbers may arise from functional stabilization or structurally canalized rearrangements, rather than from deep phylogenetic continuity. Therefore, the maintenance of 2n = 48 in cichlids should be interpreted strictly within the evolutionary framework of the group itself, without extrapolating this condition to broader macroevolutionary scales.

In genera such as *Cichlasoma*, *Geophagus*, *Heros*, *Astronotus*, *Gymnogeophagus*, *Apistogramma*, and *Crenicichla*, heterochromatin and rDNA mapping have revealed phylogenetically structured patterns useful for cryptic species delimitation (Paiz et al., 2024).

Ecologically, Neotropical cichlids contrast with marine families such as Lutjanidae or Haemulidae, which exhibit strictly conserved 2n = 48 acrocentric karyotypes due to high dispersal capacity and gene flow (Neto et al., 2011). In contrast, freshwater cichlids experience fragmentation and restricted mobility, fostering chromosomal diversification linked to behavior, ecology, and population structure (Nirchio et al., 2014).

### Intraspecific karyotypic polymorphisms

Intraspecific chromosomal polymorphisms are widely documented in Neotropical and marine fishes and encompass variation in 2n, karyotypic formula, heterochromatin distribution, NOR number or position, chromosomal banding patterns, and the presence of supernumerary elements (Galetti, 1998; Hashimoto and Porto-Foresti, 2010; Hashimoto *et al*., 2012). These changes often represent early stages of chromosomal differentiation and may contribute to restricted gene flow, local adaptation, or incipient speciation (Fuller et al., 2019; Galindo et al., 2021). For instance, chromosomal inversions in freshwater fishes have been shown to facilitate local adaptation despite high gene flow (Thorstensen et al., 2022), whereas in marine fishes with high connectivity, genetic differentiation tends to be strongly clustered within inversion-associated genomic regions, with additional differentiation peaks in low-recombining centromeric regions that do not necessarily reflect adaptive divergence (Akopyan et al., 2025).

Multiple marine and freshwater taxa exhibit pronounced structural variation within species. In *Xyrichtys novacula*, a benthic labrid with a broad Atlantic-Mediterranean distribution, 2n varies from 45 to 48 due to independent Robertsonian fusions occurring in geographically structured populations. Genomic analyses reveal three cryptic lineages with distinct fusion profiles, particularly in Caribbean populations, highlighting an ongoing divergence process shaped by limited dispersal and historical demographic structure (Nirchio et al., 2019). Likewise, *Lutjanus synagris* displays sympatric cytotypes (2n = 47-48) produced by a large metacentric chromosome derived from a recent fusion event (Nirchio *et al*., 2008). Although the mutation is not sex-linked, its low and stable frequency suggests population-level mechanisms maintaining this polymorphism.

Another illustrative case is *Rineloricaria lanceolata*, which presents up to ten sympatric karyomorphs differing in 2n (45-48) and FN (52-55). These variants arise through Robertsonian and tandem fusions, supported by the presence of interstitial telomeric sequences (ITS), and persist within a panmictic population despite substantial structural divergence (Porto et al., 2014).

Polymorphisms also include variation in NOR number or activity (see Section 4.4), differences in heterochromatin blocks, and inversion polymorphisms, which have been repeatedly documented in characids, cichlids, and siluriforms. 

B chromosomes represent one of the most thoroughly studied forms of intraspecific polymorphism in Neotropical fishes. They are especially prevalent in Characiformes, notably in the *Astyanax scabripinnis* complex, where natural populations carry up to four B chromosomes that differ in morphology, heterochromatin content and repetitive DNA composition (Moreira and Bertollo, 1991; Moreira-Filho *et al*., 2004; Castro et al., 2014). Molecular analyses show that some B chromosomes harbor functional gene copies, including the nobox gene associated with oocyte development, and may exhibit transcriptional activity (Silva *et al*., 2021). Additional evidence from other taxa, such as *Moenkhausia sanctaefilomenae*, where individuals may carry up to eight B microchromosomes, some with active NORs, further highlights the diversity of B chromosome systems (Hashimoto et al., 2012).

## Application of molecular techniques

### *In situ* hybridization (FISH)

In fish cytogenetics, the application of FISH has enabled the precise identification of centromeric sequences in *Hoplias malabaricus* (Haaf et al., 1993), repetitive sequences in the sex chromosomes of *Leporinus elongatus* (Nakayama et al., 1994), ribosomal DNA (rDNA) sites in *Salmo salar* (Pendás et al., 1993), histone gene locations in *S. salar, Salmo trutta*, and *Oncorhynchus mykiss* (Pendás *et al*., 1994), as well as telomeric sequences in *Oreochromis niloticus* (Chew et al., 2002). More recently, the development of whole chromosome painting (WCP), a variant of FISH, has allowed the identification of specific chromosomes or entire karyotypes, proving especially useful for investigating sex chromosome evolution in *Hoplias malabaricus* (Cioffi et al., 2011). These early applications paved the way for later innovations, such as chromosome painting and comparative hybridization.

### Repetitive genomic markers

Nuclear genomes are generally composed of three major classes of DNA sequences, distinguished by their repetition frequency. The first class includes unique, single-copy sequences, which do not exhibit homology with other regions of the genome. These encompass protein-coding genes, non-coding RNAs, and cis-regulatory elements that control gene expression, representing approximately 40-50% of the human genome (Liao et al., 2023). The second class consists of moderately repetitive sequences, which range from 500 to 300,000 base pairs in length, are repeated between 10 and 10⁵ times, and account for roughly 30% of all repetitive elements in the human genome. These include microsatellites, minisatellites, and various dispersed repeats, such as transposable elements. The third class comprises highly repetitive sequences, commonly referred to as satellite DNAs (satDNAs), which are typically arranged in large tandem arrays localized in pericentromeric, subtelomeric, and interstitial chromosomal regions. These sequences constitute around 8-10% of the human genome and form the core of constitutive heterochromatin, playing essential structural and functional roles in centromeres and telomeres (Liao *et al*., 2023). 

Based on their organization, repetitive sequences are broadly categorized into (1) tandem repeats, such as satellite DNAs, and (2) dispersed repeats, including transposons and retrotransposons (Charlesworth et al., 1994; Liao et al., 2023). Satellite DNAs can further be subdivided by monomer length into microsatellites (2-6 bp), minisatellites (6-100 bp), satellites (usually 150-400 bp), and macrosatellites (exceeding 1 kb) (Thakur et al., 2021). The genomic proportion of satellite DNA varies significantly across taxa, reaching over 50% in species such as the kangaroo rat (Stephan, 1987).

In fish cytogenetics, microsatellites have been successfully employed to detect chromosomal differences at high resolution. For instance, Cioffi et al. (2011) mapped microsatellites in two cytotypes of *Hoplias malabaricus* with XY and X₁X₂Y sex-chromosome systems, revealing strong hybridization signals in subtelomeric and heterochromatic regions of several autosomes, with a marked accumulation on sex chromosomes, suggesting their involvement in sex chromosome differentiation. Similarly, Utsunomia et al. (2018) reported variable patterns of microsatellite distribution among five species of *Gymnotus*, with some motifs (e.g., CA, GA, GAG) displaying band-like hybridization patterns that facilitated homologous chromosome identification. In several cases, these motifs colocalized with multigene families, indicating possible associations with gene spacer regions.

Minisatellites generally exhibit more restricted chromosomal distributions. In *Salmo salar*, for example, Pérez et al. (1999) found that two minisatellites were confined to a single chromosome pair, while a third hybridized with four distinct pairs. In *Astyanax*, the As51 minisatellite (51 bp) was first identified by Mestriner et al. (2000), and subsequent mapping by Kantek et al. (2009) across five species and multiple populations revealed preferential localization to terminal regions of subtelocentric and acrocentric chromosomes, with variable abundance (1-9 chromosome pairs).

Telomeric DNA, located at chromosome termini, consists of tandem arrays of species-specific 5-8 bp GT-rich repeats (Blackburn, 1991). The size of these arrays varies across species, from as little as 36 bp in *Oxytricha fallax* (Pluta et al., 1982) to 50-150 kb in *Mus musculus* (Zijlmans et al., 1997). In *Oreochromis niloticus*, telomeric sequences consist of the conserved vertebrate repeat (TTAGGG)n, spanning 4-10 kb in erythrocyte chromosomes (Chew et al., 2002). Notably, FISH analysis revealed additional ITS on chromosome 1, suggesting past chromosomal fusion events. This finding may help explain the reduced 2n in *Oreochromis niloticus* (2n = 44) compared to other teleost species, although the ancestral condition of the teleost karyotype remains under discussion. A review by Ocalewicz (2013) reported that telomeric arrays in fishes range from 2 to 25 kb and may shorten with age in some species. Furthermore, approximately 42% of species showed ITS, reinforcing the idea of widespread chromosomal rearrangements and possible association of telomeric motifs with dispersed elements.

Satellite DNAs have been equally informative in fish cytogenetics. Oliveira and Wright (1998) mapped the SATA (237 bp) and SATB (1,900 bp) sequences in *O. niloticus*, and observed that SATA was located in heterochromatic regions of all chromosomes, while SATB was restricted to a single chromosome pair. A broader review by Vicari et al. (2010) confirmed that fish satellite DNAs are consistently associated with heterochromatin and show lineage-specific distribution patterns.

The advent of next-generation sequencing (NGS) has substantially advanced the study of satellite DNAs. In *Astyanax paranae*, Silva *et al*. (2017) identified 45 satDNA families with monomer lengths ranging from 6 to 365 bp (median: 59 bp). Chromosomal mapping in *A. paranae*, *A. fasciatus*, and *A. bockmanni* revealed that most satellites are conserved and share similar distribution landscapes, likely reflecting recent common ancestry. However, some satellites exhibited species or chromosome-specific localization (e.g., ApaSat44-21) was exclusive to the B chromosome of *A. paranae*, while ApaSat20-18 was B-specific in *A. paranae* but present in both A and B chromosomes of the other species. The symmetrical distribution of several satellites on B chromosome arms supports the isochromosome nature of these elements, whereas asymmetry in the *A. fasciatus* BfMa B chromosome suggests an older evolutionary origin. A complementary study in Characidae by Utsunomia et al. (2017), using NGS, PCR, Sanger sequencing, and FISH, revealed high variability and diversification of satellite DNAs in this family.

Transposable elements (TEs), another major class of repetitive DNA, are mobile genetic elements classified into DNA transposons and retrotransposons, based on their transposition mechanisms. DNA transposons move via a cut-and-paste mechanism, while retrotransposons propagate through a copy-and-paste mechanism involving RNA intermediates (Kim et al., 2012). In a comparative analysis of 39 fish species, Shao et al. (2019) found TE content ranging from 5% in pufferfish to 56% in zebrafish, with a positive correlation between genome size and TE abundance. Several TE families including long interspersed nuclear elements (LINEs L1, L2) and Chicken Repeat 1 (CR1) elements, showed consistent patterns of accumulation, suggesting a role in shaping genome architecture and contributing to vertebrate evolution.

In *O. niloticus*, the LINE element CiLINE2 was cloned and characterized by Oliveira et al. (1999), who estimated approximately 5,500 copies per haploid genome. FISH mapping revealed widespread distribution across all chromosomes, with a concentration near telomeric regions. Rex retroelements, a group of non- long terminal repeat retrotransposons (LTR) originally described in *Xiphophorus* (Volff et al., 1999), are widely distributed among teleosts and typically accumulate in heterochromatic regions such as centromeres, pericentromeres, and telomeres (Carducci et al., 2018). A recent study by Souza et al. (2024) on the distribution of Rex1, Rex3, and Rex6 in *Ctenolucius* and *Boulengerella* revealed dispersed patterns with localized accumulation in both euchromatic and heterochromatic regions. According to the authors, TE presence in euchromatic areas may facilitate chromosomal rearrangements such as inversions, duplications, deletions, and translocations that underlie the karyotypic diversification observed among these genera.

### Genomic and cytogenomic comparisons

Modern cytogenomic techniques such as CGH and GISH have provided new insights into chromosome homology, genome divergence, and sex chromosome evolution. The following examples illustrate their application in diverse fish taxa. For instance, Barby et al. (2019) applied CGH to species of the family Notopteridae, which are characterized by conserved karyotype structures and 2n. Their results revealed the maintenance of overall karyotypic architecture across the family, disrupted only by specific numerical and structural rearrangements observed in *Chitala lopis* and *Papyrocranus afer*. These findings support the hypothesis of karyotype stasis in Notopteridae. However, the observed decrease in chromosomal homology over time also suggests the gradual action of intrachromosomal rearrangements, which likely contribute to the erosion of collinearity and conserved synteny.

Nagamachi et al. (2010) employed WCP probes, obtained via Fluorescence-Activated Chromosome Sorting (FACS), to investigate cytogenetic relationships within *Gymnotus carapo* sensu stricto. Using two-color FISH hybridizations in the 2n = 42 cytotype, the authors were able to distinguish multiple homologous pairs (e.g., chromosomes 1, 2, 3, 7, 9, 14, 16, 18, 19, 20, and 21). When these probes were hybridized onto metaphase spreads of the 2n = 40 cytotype, some chromosomes exhibited conserved synteny, while others showed evidence of complex rearrangements, highlighting the dynamic nature of chromosomal evolution in this group.

**Figure 4 -:**
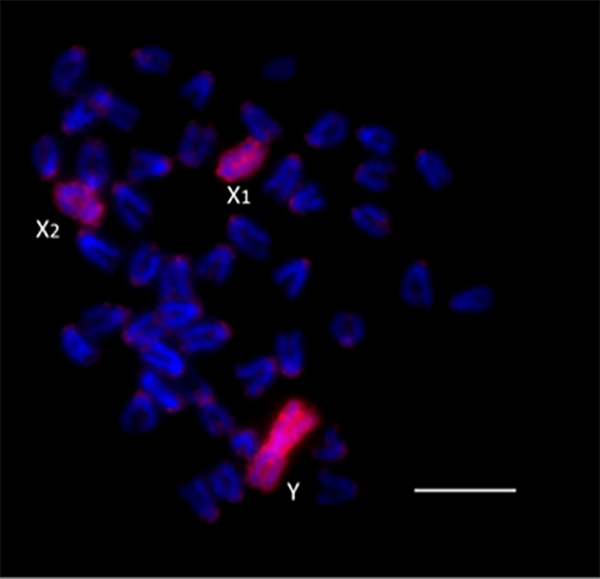
WCP with a Y-specific probe reveals homology among sex chromosomes in *Oplegnathus punctatus*. A Y-specific probe (red), obtained from male chromosomes of *O. punctatus*, was hybridized onto metaphase spreads of the same species. The probe fully labeled the Y, X₁, and X₂ chromosomes, indicating extensive sequence homology among these elements and supporting their common evolutionary origin. This cytogenetic pattern confirms the presence of a multiple sex chromosome system (X₁X₁X₂X₂ in females and X₁X₂Y in males), likely derived from chromosomal rearrangements involving ancestral autosomes. Chromosomes were counterstained with DAPI (blue). Scale bar = 10 µm. Image kindly provided by Dr. Marcelo de Bello Cioffi (Universidade Federal de São Carlos, Brazil).

Techniques such as microdissection allowed the isolation of single chromosomes that can be marked with different fluorescent compounds and hybridized to locate these chromosomes in different species or in males and females of the same species. WCP in *Oplegnathus punctatus*, confirmed homology among sexes (See Figure 4 for details). 

**Figure 5 -:**
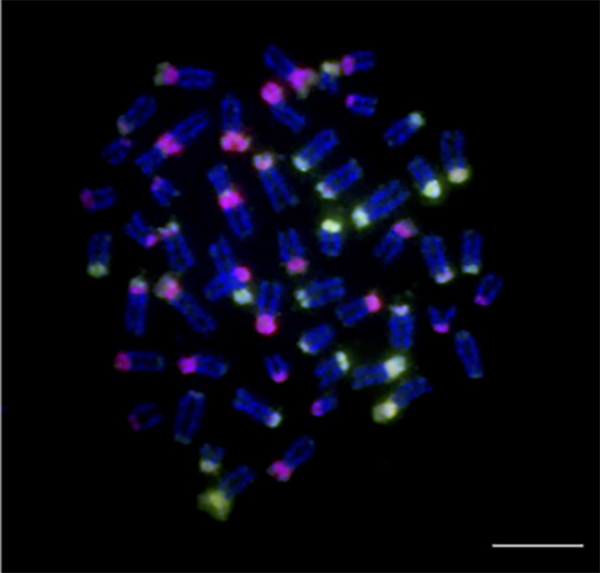
CGH reveals interspecific genomic divergence between South American arowanas (*Osteoglossum ferreirai* and *O. bicirrhosum*). Total genomic DNA of *O. ferreirai* (red) and *O. bicirrhosum* (green) were co-hybridized onto metaphase chromosomes of *O. ferreirai.* Overlapping signals appear in yellow, indicating conserved repetitive DNA regions, while distinct red or green signals denote species-specific sequences. Chromosomes were counterstained with DAPI (blue). This CGH approach illustrates genomic divergence despite overall karyotypic conservation in basal teleosts. Scale bar = 10 µm. Image kindly provided by Dr. Marcelo de Bello Cioffi (Universidade Federal de São Carlos, Brazil).

A particularly illustrative case of interspecific CGH involves South American arowanas (*Osteoglossum ferreirai* and *O. bicirrhosum*). In this experiment, total genomic DNA from *O. ferreirai* (labeled in red) and *O. bicirrhosum* (in green) were simultaneously hybridized onto metaphase chromosomes of *O. ferreirai*, counterstained with DAPI (blue). The resulting hybridization pattern revealed conserved regions (yellow fluorescence from signal overlap) and divergent genomic regions (distinct red or green signals), even though both species exhibit similar karyotypes. This approach underscores the value of CGH in revealing fine-scale differences in repetitive DNA and supports its use in exploring genomic divergence among basal teleosts (Figure 5).

In the genus *Triportheus*, all species share a ZW sex chromosome system, in which the Z chromosome is consistently the largest element in the karyotype, while the W chromosome displays varying degrees of differentiation from nearly homomorphic to highly heteromorphic forms (Yano et al., 2016). To assess whether W chromosome differentiation reflects phylogenetic divergence and to test the hypothesis of a shared origin of the ZW system, the authors applied CGH and cross-species WCP. Their results indicated a common origin of the ZW sex chromosome system in *Triportheus*, evidenced by the conserved nature of the Z chromosome and marked divergence of the W chromosome across species.

Chromosome painting has also provided important insights into the origin of B chromosomes. In *Moenkhausia sanctaefilomenae*, Scudeler et al. (2015) demonstrated that a particular B chromosome shared DNA sequences with several A chromosomes in all populations analyzed, supporting an intraspecific origin. However, other B chromosome variants present in the same individuals showed no hybridization signals, suggesting independent origins and the coexistence of distinct B chromosome types within a single species.

Building upon the cytogenomic approaches detailed above, comparative analyses using CGH and WCP have provided deeper insights into chromosomal homologies, lineage-specific rearrangements, and genome divergence. These techniques have proven especially useful in identifying cryptic species, tracking sex chromosome differentiation, and elucidating chromosomal evolution across taxa.

## Sex chromosomes and sex determination systems

Sex determination in teleost fishes encompasses one of the widest ranges of chromosomal architectures known among vertebrates. Broad comparative and cytogenomic studies demonstrate that teleosts exhibit extraordinary diversity in sex-determination mechanisms, including classical XX/XY, XX/X0, and ZZ/ZW systems, as well as numerous neo-sex and multiple-chromosome configurations that have arisen independently across lineages (Mank and Avise, 2009; Arai, 2011; Cioffi et al., 2017; Sember et al., 2021). Cytogenetic evidence indicates that teleosts possess nine cytogenetically distinct sex-chromosome systems, reflecting repeated origins and transitions among alternative modes of sex determination (Sember *et al*., 2021). These nine systems include the standard XX/XY and ZZ/ZW configurations, their derived forms resulting from Y or W chromosome loss (XX/X0 and ZZ/Z0), and five types of multiple systems generated through Robertsonian fusions, centric fissions, or compound rearrangements (X₁X₁X₂X₂/X₁X₂Y; XX/XY₁Y₂; X₁X₁X₂X₂/X₁Y₁X₂Y₂; ZZ/ZW₁W₂; and Z₁Z₁Z₂Z₂/Z₁Z₂W). Examples of each configuration across Neotropical lineages are synthesized in Table 1.

**Table 1 -:** Documented examples of sex-chromosome systems in Neotropical teleosts and their underlying chromosomal mechanisms.

Group / Family	Species / Complex	Sex-chromosome system	Mechanism / Cytogenetic features	Evidence / Notes	Reference
Characiformes (Triportheidae)	*Triportheus* spp.	ZZ/ZW	Conserved sex chromosome system across species	Stable female heterogamety	Yano et al., 2017
Gymnotiformes (Gymnotidae)	*Gymnotus bahianus*	XX/XY₁Y₂	Multiple sex chromosome system involving Y-derived chromosomes	Different 2n in males (37) and females (36)	Almeida et al., 2015
Gymnotiformes (Gymnotidae)	*Gymnotus pantanal* / *Gymnotus* sp.	X₁X₁X₂X₂ / X₁X₂Y	Robertsonian fusion generating neo-Y	Large AT-rich neo-Y; loss of GC-rich regions	Sánchez et al., 2004; Silva et al., 2011
Siluriformes (Doradidae)	*Tenellus trimaculatus*	ZZ/ZW	Differentiated ZW chromosomes	Early sex chromosome differentiation	Takagui et al., 2017
Siluriformes (Doradidae)	*Platydoras armatulus* / *Ossancora punctata*	B chromosomes interacting with sex regions	Supernumerary elements associated with sex	Potential sex-linked B chromosomes	Takagui et al., 2017
Characiformes (Anostomidae)	*Leporinus reinhardti*	ZZ/ZW	Accumulation of repeated DNAs on the W chromosome	W highly heterochromatic; microsatellite and 18S rDNA-associated differentiation	Cioffi and Bertollo, 2012
Gymnotiformes (Sternopygidae)	*Eigenmannia aff. desantanai*	ZZ / ZW₁W₂	W-multiplicity (first case in Gymnotiformes)	Complex female heterogamety	Araújo et al., 2023
Gymnotiformes (Sternopygidae)	*Eigenmannia virescens*	XX/XY and ZZ/ZW (coexistence)	Independent origins within species complex	Rapid turnover within genus	Almeida-Toledo et al., 2002

Among the multiple systems documented in teleosts, several configurations are particularly well characterized. The X₁X₁X₂X₂/X₁X₂Y system typically results from a Robertsonian fusion between the ancestral Y chromosome and an autosome, producing a neo-Y chromosome with restricted recombination. This arrangement has been documented in *Hoplias malabaricus*, *Oplegnathus punctatus*, and multiple species of *Gymnotus*. Conversely, a population identified as *Gymnotus bahianus* was reported to exhibit an XX/XY₁Y₂ multiple sex chromosome system, in which males possess two Y-derived chromosomes (Almeida et al., 2015).

Female-heterogametic multiple systems also occur, although less frequently. The ZZ/ZW₁W₂ system produced through structural diversification of the W chromosome is reported in *Apareiodon affinis* and represents a rare example of W-chromosome multiplication. Similarly, *Eigenmannia* aff. *desantanai* exhibits a ZW₁W₂/ZZ system, constituting the only confirmed case of multiple-W heterogamy in Gymnotiformes (Araújo et al., 2023). Independent origins and turnover of sex chromosome systems are also evident in *Eigenmannia virescens*, in which both XX/XY and ZZ/ZW systems have been reported (Almeida-Toledo et al., 2002). In Characiformes, the Z₁Z₁Z₂Z₂/Z₁Z₂W system found in *Megaleporinus* species exemplifies the role of chromosomal fusion and heterochromatin accumulation in generating complex sex chromosomes (Bello Cioffi et al., 2012; Cioffi *et al*., 2017).

## Cytotaxonomy, species delimitation, and phylogeny

Cytogenetic analyses have played a pivotal role in delimiting species and resolving taxonomic ambiguities in Neotropical fishes, especially in groups with high morphological plasticity, cryptic diversity, or controversial classifications. As one of the most biodiverse freshwater ichthyofaunas in the world, Neotropical fish lineages offer fertile ground for integrative taxonomic approaches that combine cytogenetics with molecular phylogenies.

Recent cytogenetic and genomic studies have revealed remarkable chromosomal diversity within the subfamily Corydoradinae. Seminal work by Oliveira et al. (1993) provided one of the first comprehensive cytogenetic analyses of *Corydoras* species, documenting extensive variation in 2n, karyotype structure, and nuclear DNA content across multiple taxa. At the time, all species were grouped within a single genus (*Corydoras*), and the subfamily included only three recognized genera. Based on their findings, the authors hypothesized the existence of several independent evolutionary lineages within the group well before molecular tools were available.

More recently, cytogenetic analyses such as those by Barbosa et al. (2017) and Rocha et al. (2022) have reinforced these early observations, revealing additional chromosomal variation linked to population divergence and species boundaries. Importantly, integrative approaches combining cytogenetics, molecular phylogenetics, and morphological data have led to a major taxonomic revision: the subfamily Corydoradinae is now composed of seven monophyletic genera (Dias et al., 2025). This reclassification reflects a more accurate understanding of the evolutionary relationships within the group and underscores the pivotal role of cytogenomics in resolving taxonomic complexity.

In a similar context, the chromosomes of *Pyrrhulina australis* and *Pyrrhulina* aff. *australis* were studied using CGH and WCP. Although both taxa share the same diploid number (2n = 40), interspecific CGH experiments revealed species-specific hybridization patterns, indicating ongoing genomic divergence between the two forms (Moraes et al., 2019). These findings highlight the usefulness of cytogenetic approaches for detecting cryptic genomic differentiation in closely related fish taxa. 

In another example of cytogenomic inference, a DNA fragment isolated from the heterochromatic region of the W chromosome of *Apareiodon ibitiensis* designated as Wap, was microdissected and used as a probe for in situ hybridization across nine species of the family Parodontidae. The distribution of this repetitive sequence revealed shared hybridization signals among species and provided insights into the evolutionary differentiation of the ZZ/ZW sex chromosome system in the family. These results suggest that the WAp sequence played a role in the genomic restructuring and diversification of sex chromosomes in Parodontidae (Schemberger et al., 2011). Phylogenetic inferences derived from cytogenetic characters supported this model of sex chromosome differentiation and revealed monophyletic clusters among closely related species, suggesting a shared evolutionary origin of the ZZ/ZW system in Parodontidae.

## Contribution to species delimitation

Cytogenetic analyses have played a critical role in uncovering cryptic diversity within morphologically defined taxa, contributing substantially to species delimitation. In the Iguazú River basin, karyotypic studies in *Astyanax* have revealed the presence of endemic cytotypes, including unnamed forms such as *Astyanax* sp. B, C, and D, that differ in nucleolar organizer region (NOR) positions, heterochromatin distribution, and FN. These cytogenetic differences suggest reproductive isolation and support the occurrence of cryptic speciation within the basin (Kantek et al., 2007, 2009).

A notable case of hidden diversity is the *Hoplias malabaricus* species complex, which comprises at least seven distinct cytotypes distributed across South America. In the Iguazú River, cytotypes A and B appear to reflect ancient colonization events that predate the hydrographic isolation of the basin (Dergam et al., 1998; Bertollo et al., 2000). 

The fish species *Hoplias malabaricus* represents a well-known species complex characterized by remarkable karyotypic diversity, including homomorphic and highly differentiated sex-chromosome systems across its karyomorphs. Using an integrated cytogenetic approach based on CGH and analyses of repetitive DNA distribution, Sember et al. (2018) investigated genomic differentiation among karyomorphs of *Hoplias malabaricus*, including karyomorph F. Their results revealed a nascent XX/XY sex chromosome system in which the Y chromosome carries a male-specific interstitial heterochromatic block enriched with microsatellite motifs and retrotransposons. The accumulation of repetitive sequences in this region suggests an early stage of sex chromosome differentiation and highlights the dynamic chromosomal evolution occurring within the *H. malabaricus* species complex.

Similarly, an integrative study combining cytogenetic and molecular data in *Apareiodon* (Parodontidae) populations from the Aripuanã River revealed a previously undescribed species characterized by a ZZ/ZW sex chromosome system, multiple rDNA sites, and a unique distribution of repetitive DNA sequences (pPh2004 and WAp). This distinct chromosomal profile was supported by significant divergence in the Cytochrome c oxidase subunit I (COI) barcode, allowing the recognition of a separate Molecular Operational Taxonomic Unit (MOTU) (Santos et al., 2019). In this case, chromosomal markers were essential for the diagnosis of a cryptic lineage that would likely have gone undetected using morphological criteria alone.

### Cases of cryptic or misidentified species

Several morphologically homogeneous fish groups have been resolved into multiple cryptic species through cytogenetic data. In the genus *Rhamdia*, taxa such as *R. branneri*, *R. voulezi* and *R. quelen* were previously treated as congeners. However, distinct karyotypes, including B chromosomes and NOR variations, support their separation (Abucarma and Martins-Santos, 2001). In *Pimelodus ortmanni*, the cytogenetics and isoenzymatics data exposed the presence of a distinct, undescribed species (Borin and Martins-Santos, 2004).

A comprehensive molecular study of the Stevardiinae genera *Bryconamericus*, *Eretmobrycon*, *Knodus* and *Hemibrycon* revealed that nearly 50% of the specimens examined were misidentified in ichthyological collections. Species delimitation using three DNA based models (GMYC, PTP, ABGD) confirmed multiple new lineages, clarified synonymies, and uncovered taxonomic errors, highlighting the need for integrative taxonomy (García-Melo et al., 2019).

In the genus *Brachygalaxias* (Galaxiidae), karyotypic differences such as chromosomal morphology, heterochromatin patterns and NOR distribution distinguished *B. gothei* from *B. bullocki*, despite morphometric overlap. The sterility and intermediate karyotype of the hybrids further supported their reproductive isolation, validating *B. gothei* as a separate species (Cuevas et al., 1999).

### Integration with molecular phylogenies

Combining cytogenetic data with molecular phylogenies strengthens evolutionary and biogeographical interpretations. In *Hoplias malabaricus*, cytotype distribution aligns with mitochondrial COI and Random Amplified Polymorphic DNA (RAPD) divergence, delineating isolated evolutionary lineages across river basins (Dergam et al., 1998; Bertollo et al., 2000). Likewise, lineages of *Apareiodon* with distinct sex chromosomes and rDNA profiles are congruent with molecular clades (Santos et al., 2019).

In Stevardiidae, García-Melo et al. (2019) proposed an integrative workflow incorporating DNA barcoding and morphological reassessment. Their approach resolved numerous species complexes, reassigned genera, and highlighted the extensive cryptic diversity masked by homoplastic morphological traits, particularly in *Eretmobrycon emperor*, where at least six distinct lineages were identified based on COI and geographic segregation data.

These integrative frameworks not only refine taxonomy, but also help to reconstruct historical biogeographic patterns, such as vicariance or fluvial capture events, especially when chromosomal signatures coincide with molecular divergence, as observed in *Gymnotus species* (Milhomem et al., 2008).

## Challenges and perspectives

Despite significant advances in cytogenetic characterization of Neotropical fishes, fundamental challenges remain. Understanding the mechanisms driving chromosomal diversification is essential, especially in groups with miniaturized bodies and cryptic morphologies, such as the genus *Pyrrhulina* (Lebiasinidae). Recent studies integrating classical cytogenetic techniques with modern molecular tools such as CGH and WCP along with population genomic data have revealed remarkable karyotypic variability in *Pyrrhulina*, including the occurrence of multiple sex-chromosome systems (e.g., the X₁X₂Y system in *P. semifasciata*) and significant differences in repetitive DNA content (Moraes et al., 2019; Ahmad et al., 2022).

These chromosomal rearrangements, particularly those associated with the sex chromosomes, could act as effective reproductive barriers. Genomic evidence based on single nucleotide polymorphisms (SNPs) indicates that genetic differentiation among *Pyrrhulina* species is consistent with their chromosomal differences and cannot be explained exclusively by geographic distribution, reinforcing the hypothesis that chromosomal evolution plays a key role in speciation (Ferreira et al., 2022).

Likewise, it is a priority to address information gaps in difficult to study groups such as the genus *Astroblepus* (Siluriformes: Astroblepidae), widely distributed in high Andean mountain systems. This genus presents important challenges in its systematics due to its remarkable phenotypic plasticity and often ambiguous or contradictory historical taxonomic descriptions. Variability in body shape, fin size, and coloration patterns have contributed to the taxonomic complexity of the group (Ochoa et al., 2020). In addition, the limited availability of specimens and difficult access to their habitats have made it difficult to resolve their phylogenetic relationships and species delimitation. In this context, the application of next-generation cytogenetic and genomic approaches could provide key evidence to clarify their taxonomic status, identify cryptic lineages, and understand patterns of chromosomal evolution in extreme environments (Nirchio et al., 2025).

In a broader context, cytogenetic studies remain limited for many fish groups characterized by high biodiversity, restricted geographic distribution, or challenging access. However, the increasing adoption of high-throughput sequencing technologies and integrative chromosomal approaches offers an unprecedented opportunity to reconstruct the evolutionary history of fish karyotypes at both macrostructural and microstructural levels (Rhie et al., 2021; Ahmad et al., 2022). The generation of high-quality genome assemblies, combined with cytogenetic mapping, will be essential to investigate chromosomal rearrangements, the origin and evolutionary turnover of sex chromosomes, and the distribution dynamics of repetitive DNA elements.

A key challenge moving forward will be the application of these cytogenomic approaches across a broader range of taxonomic groups and geographic regions, while simultaneously incorporating ecological, environmental, and demographic variables. Understanding the contribution of chromosomal architecture to reproductive isolation and lineage diversification will not only enhance taxonomic resolution, but also inform conservation strategies for endemic and threatened species.

To advance the field, we recommend prioritizing to (i) integrate cytogenetic and genomic data across phylogenetically and ecologically diverse lineages; (ii) development of species-specific probes for fine-scale chromosomal mapping; and (iii) long-term population studies to assess the role of chromosomal systems in evolutionary and ecological processes. These strategies will be key to achieving a more comprehensive understanding of karyotype evolution and its role in generating and maintaining biodiversity in the Neotropical region.

## Conclusions

Over the past five decades, cytogenetic research has profoundly reshaped our understanding of chromosomal diversity, evolution, and speciation in Neotropical fishes. Initially centered on descriptive karyotype characterization, the field has evolved toward a highly integrative discipline that now incorporates molecular cytogenetics, genomics, and phylogenetics. This shift has not only increased the resolution with which chromosomal features can be analyzed, but has also uncovered extensive cryptic diversity, lineage-specific rearrangements, and dynamic sex chromosome evolution across multiple fish lineages.

The implementation of advanced tools such as FISH, CGH, WCP has been instrumental in revealing genomic compartments, repetitive DNA distribution, and interspecific chromosomal synteny. These tools have proven essential for cytotaxonomy and for refining species delimitations, particularly in morphologically conserved or highly diverse groups like *Astyanax*, *Hoplias*, *Corydoras*, and *Pyrrhulina*. Furthermore, the study of sex chromosomes and repetitive sequences has shed light on the mechanisms driving chromosomal diversification and reproductive isolation.

Despite the progress made, a significant portion of Neotropical ichthyofauna remains cytogenetically unexplored. Continued efforts to characterize these taxa using high-resolution and integrative approaches are critical not only for completing evolutionary frameworks, but also for informing conservation strategies in biodiversity hotspots increasingly threatened by anthropogenic pressures.

Biogeographic patterns across the Neotropics offer essential context for interpreting chromosomal evolution in freshwater fishes. The region’s complex geological and hydrological history (marked by drainage rearrangements, episodic basin isolation and reconnection, and pronounced ecological gradients) has repeatedly promoted demographic fragmentation and facilitated the fixation of structural rearrangements. In contrast, high connectivity and large effective population sizes in marine environments tend to stabilize karyotypic configurations, contributing to the recurrent emergence of similar diploid numbers such as 2n = 48. These contrasts indicate that karyotypic diversity in Neotropical fishes is tightly linked to historical landscape dynamics and reinforce that conserved acrocentric configurations in marine lineages likely represent convergent or stabilized states rather than ancestral conditions.

In this context, the prevalence of acrocentric 2n = 48 karyotypes in marine lineages should be interpreted cautiously and may reflect recurrent or stabilized chromosomal states rather than direct evidence of ancestral retention. Recognizing this pattern helps refine interpretations of chromosomal evolution of teleosts and avoids overly linear evolutionary inferences.

In sum, cytogenetics has become a cornerstone of Neotropical fish biology, offering unparalleled insights into genomic architecture and evolutionary processes. As technological advances continue to expand the toolkit of cytogeneticists, future studies will likely reveal even deeper layers of chromosomal innovation, reinforcing the relevance of this field for evolutionary biology, taxonomy, and conservation.

## Data Availability

This review does not include original datasets. All data discussed are publicly available and properly cited in the reference list.
